# Effectiveness of 675-nm Wavelength Laser Therapy in the Treatment of Androgenetic Alopecia Among Indian Patients: Clinical Experimental Study

**DOI:** 10.2196/60858

**Published:** 2024-09-23

**Authors:** BS Chandrashekar, Oliver Clement Lobo, Irene Fusco, Francesca Madeddu, Tiziano Zingoni

**Affiliations:** 1 Cutis Academy of Cutaneous Sciences Bengaluru India; 2 El.En. Group Calenzano Italy

**Keywords:** androgenetic alopecia, AGA, 675-nm laser, Indian patients, hair restoration, effectiveness, laser therapy, therapy, treatment, Indian, patients, patient, India, hair loss, hair, laser stimulation, hair density

## Abstract

**Background:**

Androgenetic alopecia (AGA) is the most prevalent cause of hair loss around the world.

**Objective:**

The purpose of this study was to evaluate the efficacy of laser stimulation with a 675-nm wavelength for the treatment of AGA in male and female Indian patients.

**Methods:**

A total of 20 Indian healthy patients aged 23-57 years who presented a grade of alopecia stage I to stage V underwent one single pass with a 675-nm laser to the scalp area twice a week for a total of 8 sessions, followed by once a week for 4 sessions and once in 2 weeks for 2 sessions. There are 14 laser treatments in total. Macro- and dermatoscopic images have been acquired at T0 (baseline) and T1 (4 months). The vertex, frontal, and parietal areas of the scalp were evaluated. Many parameters were analyzed including hair count and hair density of terminal; mean thickness; vellus follicles; total follicular units; units with 1 hair, 2 hairs, 3 hairs, 4 hairs, and >4 hairs; unit density; and average hair/unit.

**Results:**

The macroimages and dermatoscopic evaluations showed good improvement over the entire treated area, with a clear increase in the number of hairs and hair thickness. General parameters such as hair count and hair density showed a percentage increase of around 17%. The hair mean thickness parameters showed a significant (*P*<.001) percentage increase of 13.91%. Similar results were obtained for terminal and vellus hair: terminal hair count and hair density significantly (*P*=.04 and *P*=.01, respectively) increased by 17.45%, vellus hair count increased by 16.67% (*P*=.06), and the density of vellus hair increased by 16.61% (*P*=.06).

**Conclusions:**

The study findings demonstrate that the 675-nm laser system improved AGA in Indian patients, facilitating the anagen phase and improving hair density and other positive hair parameters.

## Introduction

Worldwide, androgenetic alopecia (AGA) represents the most common cause of hair loss. Up to 70% of men and 40% of women are affected, primarily affecting the frontal and parietal regions of the scalp.

AGA can affect all races, but the prevalence rates vary. Prevalence is considered to be highest in White men [1,2]. Male AGA phenotypic variants are graded using the universally accepted modified Norwood-Hamilton classification. AGA grade scores range from I to III for men according to the Norwood-Hamilton scale [3] and from I to II for women according to the Ludwig scale [4].

According to statistics, at least 50% of male individuals by age 50 years and a similar proportion of female individuals by age 60 years will experience this medical disorder [5]. A new community-based study in Singapore found that 87% of Indian individuals are affected, compared to more than 61% of Chinese individuals [6].

In the Indian context, a population-based study of 1005 patients showed a 58% prevalence of AGA in male individuals aged 30-50 years [7], and a large study also reported that stage II was the most common presentation of AGA in the Indian population [8]. Another study conducted on the Indian population found stage II and III as the most common presentations [9].

Affected individuals experience psychological and emotional consequences [10]. Androgens, including testosterone and its derivatives, frequently cause AGA in genetically predisposed individuals [11]. It manifests as a gradually shorter anagen phase in terminal hair follicles (HFs) and a final hair cycle transition from terminal to intermediate to vellus hair on the scalp in a characteristic pattern [12].

Receptor activation pathways play a role in the development of AGA in mini complex organs. In this setting, the increased sensitivity to androgenic hormones in individuals with AGA negatively impacts the Wnt/β-catenin signaling pathway, which is crucial for stimulating the anagen phase [13]. The HFs in areas affected by AGA have an oval dermal papilla, a very thin matrix, low levels of melanin in the catagen phase, and often exhibit recurring nondestructive perifollicular microinflammation along with the deposition of mastocytes, macrophages, and lymphocytes. This condition leads to progressive follicular fibrosis and thickening of the fibrocollagenous sheath [14].

Currently, the US Food and Drug Administration has approved three therapies for AGA: topical minoxidil, topical finasteride, and lower-level laser therapy. However, some of these treatments are associated with several side effects and unsatisfactory outcomes.

Minoxidil is associated with a number of adverse reactions, including pruritus, scalp irritation, irritant and allergic contact dermatitis, cardiovascular system symptoms/signs in a dose-dependent manner, and facial hypertrichosis [15-17]. Although primarily utilized in dermatologic applications, finasteride has been associated with hepatic dysfunction, unilateral breast enlargement and palpitations, libido reduction, head pain, fever, sexual dysfunction, and neuropsychiatric side effects [18-20]. In clinical practice, various medication treatments like progesterone, azelaic acid, zinc salts, flutamide, dutasteride, and spironolactone, as well as invasive techniques like platelet-rich plasma, scalp microneedling, and hair transplantation, are commonly employed, but they have not demonstrated conclusive outcomes or promising prospects [16]. For this reason, research into specific treatments for AGA is needed.

Devices based on light-emitting diodes (LEDs) represent the most innovative and secure therapy solution for a range of diseases, such as aging, dysfunctional hair development, and skin inflammatory illnesses [21]. Numerous research studies have documented the efficacy of photodynamic treatment (PDT) in the management of hair loss [22]. When compared to alternative treatments, PDT provides patients with numerous advantages. It has a good safety profile and is noninvasive, affordable, and convenient for patients. Pharmacotherapy and other treatment methods can be combined or substituted with PDT [23].

The most common light source mentioned in studies is represented by low-level laser therapy [22,24-26]. A recently published study [27] conducted on Indian patients demonstrated that the application of low-level laser therapy in combination with a minoxidil topical solution can successfully raise the percentage of patients who recover from AGA and enhance patients’ satisfaction with treatments received for hair regrowth.

The efficacy of LED therapy with visible light has additionally been accepted as a valid adjuvant treatment in the recalcitrant form of alopecia areata [28]. Furthermore, real effectiveness in treating hair loss has recently been shown with LED therapy, especially with treatments that use red and infrared wavelengths.

Palma and colleagues [29] reported the first case in which photobiomodulation therapy with a continuous wavered laser (660 nm) was successfully used as monotherapy for AGA. It has been demonstrated that the anagen phase, which is the active growth phase of HFs, can be stimulated with great success at a wavelength of 660 nm. Studies conducted in vitro demonstrate how red light can prolong the anagen phase and postpone the catagen transition [30]. Specifically, near-infrared light has been used to stimulate cell proliferation and differentiation of stem cells [31]. The effect was established in vitro by the degree of expression of Ki-67, an indicative biomarker of cell proliferation in the hair matrix [32].

The recent published investigation of Sorbellini et al [33] assessed the efficacy of 675-nm laser emissions for the management of androgenetic alopecia in female and male patients. The results showed a significant increase in the density of the hair shafts, resulting in a 60% reduction of the miniaturization process in the treated areas without side effects.

Based on these scientific findings, the purpose of this study was to evaluate the efficacy of laser stimulation with a 675-nm wavelength for the treatment of AGA in male and female Indian patients, which currently has limited research.

## Methods

### Recruitment

From August 2023 to March 2024, a total of 20 Indian healthy patients (7 female and 13 male) aged 23-57 years and who presented with Alopecia stage I to stage V were enrolled. AGA severity ranges from stage I to V for men according to the Norwood-Hamilton scale and from stage I to III for women according to the Ludwig scale.

AGA was diagnosed based on dermatologic and clinical examinations. The following exclusion criteria were used: topical or systemic treatments for AGA in the 3 months preceding the study, systemic or cutaneous comorbidities on an autoimmune basis or involving connective tissue, and pregnancy.

### Ethical Considerations

The study received ethics approval from the Cutis Institutional Ethics Committee (CIEC) on July 14, 2023. It was conducted in accordance with the Declaration of Helsinki on ethical principles for medical research involving human subjects. All data utilized in this study were managed securely and deidentifed to ensure the rights and privacy of the participants. Informed consent was obtained from all patients involved in the study. This research received no external funding. No compensation was provided to participants for their time participating in this research.

### Device Description

The RedTouch laser (Deka M.E.L.A, Calenzano, Italy) device was used. The study device emits a wavelength of 675 nm, and it is equipped with a 13 × 13 mm scanning system able to generate fractional microzones with a width of 0.7 mm (DOT area) of subablative and selective thermal damage on the skin. The presence of an integrated skin cooling system and the possibility to add a contact sensor minimizes downtime and possible adverse effects, and protects the epidermal layer. This laser can have different effects on the skin. At low energies, it creates a reversible thermal area that biostimulates to a depth of 3-6 mm while increasing the energy results in the formation of a coagulation column to a depth of 0.5-1 mm and deeper reversible heating.

### Study Protocol and Clinical Photographic Assessment

All patients underwent one single pass of the 675-nm laser to the scalp area twice a week for a total of 8 sessions, followed by once a week for 4 sessions and once every 2 weeks for 2 sessions. The duration time of each session was 20 minutes, and the following parameters were selected: power 1 W, dwell time 100 ms, stack 1, spacing of 1000 µm, and a cooling temperature set at 15 °C. At the end of the treatment protocol, patients completed 14 laser sessions.

Macro- and dermatoscopic images were acquired at T0 (baseline) and T1 (4 months). For the dermatoscopy analysis, the Fotofinder device (FotoFinder Trichoscale System, GmbH 1000, Bad Birnbach, Germany) was used at T0 (baseline) and T1 (4 months). The dermatoscopic analysis of each patient was compared at baseline and after 4 months to quantitatively assess the hair. The vertex, frontal, and parietal areas of the scalp were evaluated.

Many parameters were analyzed including hair count and hair density of terminal; mean thickness; vellus follicules; total follicular units; units with 1 hair, 2 hairs, 3 hairs, 4 hairs, or more than 4 hairs; unit density; and average hair/unit.

Hair count and hair density indicate the number of hairs in the analyzed area without distinguishing between terminal hairs and vellus (which are analyzed with the next two parameters). Finally, the total number of follicular units, the density of the units, the number of hairs per follicular unit, and the average number of hairs per unit were analyzed. An increase in the number of follicular units, mean thickness, and number of hairs per follicular unit was considered a positive outcome at the end of 4 months (T1).

### Side Effects

Possible side effects such as hair burn, blistering, scarring, burns, hypopigmentation, or hyperpigmentation that may result from the use of given energy levels are monitored for the entire treatment period.

### Pretreatment Procedure

To prevent light reflections and to keep the handpiece’s cooling temperature uniform, the patient’s skin and hair were soaked with water prior to the treatment. To increase patient comfort and allow the patient to leave easily after the treatment, hair gel was not used.

### Statistical Analysis

All clinical data were reported as means and SDs. The statistical analysis was carried out using a Student *t* test (1-tailed). A *P* value of .05 was selected as the cutoff for significance.

## Results

Macro- and dermatoscopic images of patients’ scalp areas taken at T0 and T1 were quantitatively evaluated (Table 1).

**Table 1:** Mean clinical parameters for 20 patients at baseline (T0) and follow-up (T1), and the percentage difference

	T0, mean (SD)	T1, mean (SD)	Percentage difference (%)	*P* value
Hair count^a^	104.62 (23.71)	122.60 (30.19)	+17.19	*<*.001
Hair density (cm^2^)^a^	115.82 (26.24)	135.72 (33.42)	+17.18	<.001
Hair count terminal^a^	69.58 (18.37)	81.72 (19.00)	+17.45	.04
Hair count vellus	35.04 (19.83)	40.88 (16.67)	+16.67	.06
Hair density terminal (cm^2^)^a^	77.03 (20.33)	90.47 (21.03)	+17.45	.01
Hair density vellus (cm^2^)	38.81 (21.93)	45.26 (23.58)	+16.61	.06
Mean thickness (mm)^a^	0.05 (0.01)	0.06 (0.01)	+13.91	<.001
Total follicular units^a^	91.36 (11.69)	100.85 (12.45)	+10.39	<.001
Units (1 hair)	50.71 (3.44)	51.90 (4.34)	+2.35	.16
Units (2 hairs)^a^	27.62 (5.37)	32.01 (5.29)	+15.89	<.001
Units (3 hairs)^a^	9.89 (3.33)	11.71 (3.60)	+18.40	.01
Units (4 + >4 hairs)^a^	3.14 (2.21)	5.21 (2.09)	+69.92	<.001
Units density (cm^2^)^a^	101.24 (13.10)	109.64 (16.03)	+8.30	.004
Average hair/unit^a^	1.61 (0.12)	1.71 (0.10)	+5.97	<.001

^a^Statistically significant clinical data (*P*<.05).

**Figure 1:**
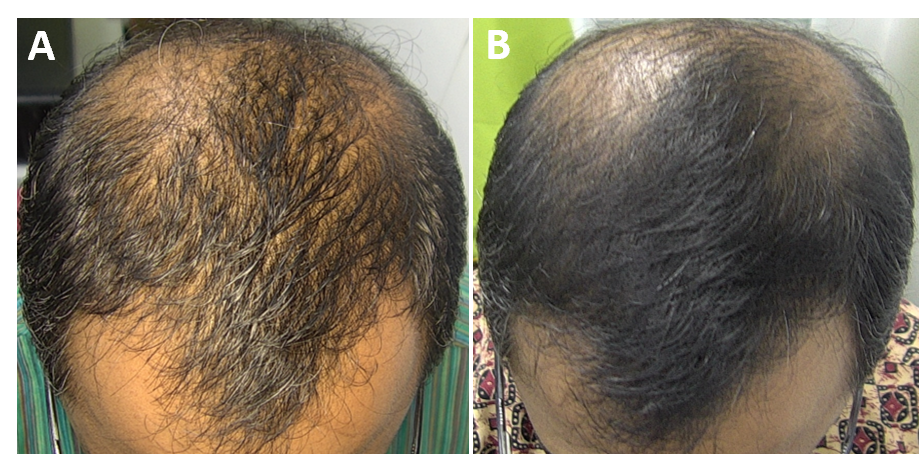
Scalp area of Indian male patient before (A) and after 14 sessions with 675-nm laser (B). A clinical improvement and restoration of the scalp’s central hairline hairs from baseline (T0) to the 4-month follow-up (T1) were observed.

**Figure 2:**
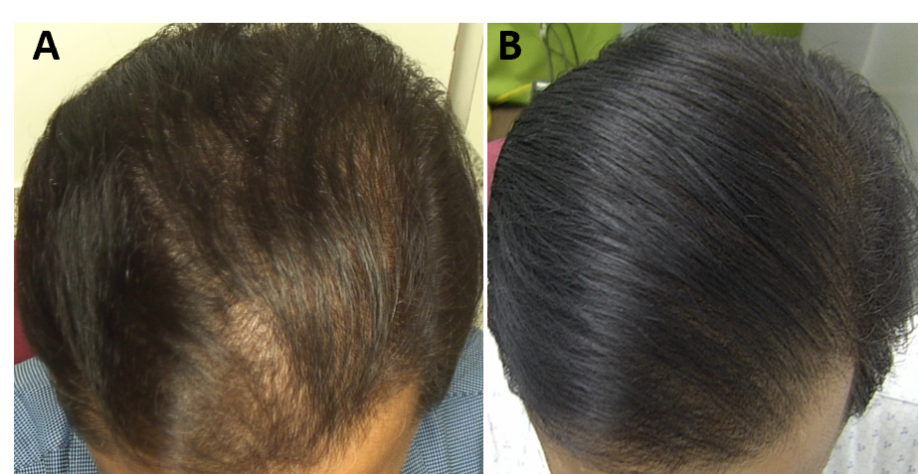
Scalp area of Indian male patient before (A) and after 14 sessions with 675-nm laser (B). A clinical improvement and restoration of the scalp’s central hairline hairs from baseline (T0) to 4-month follow-up (T1) were observed.

**Figure 3:**
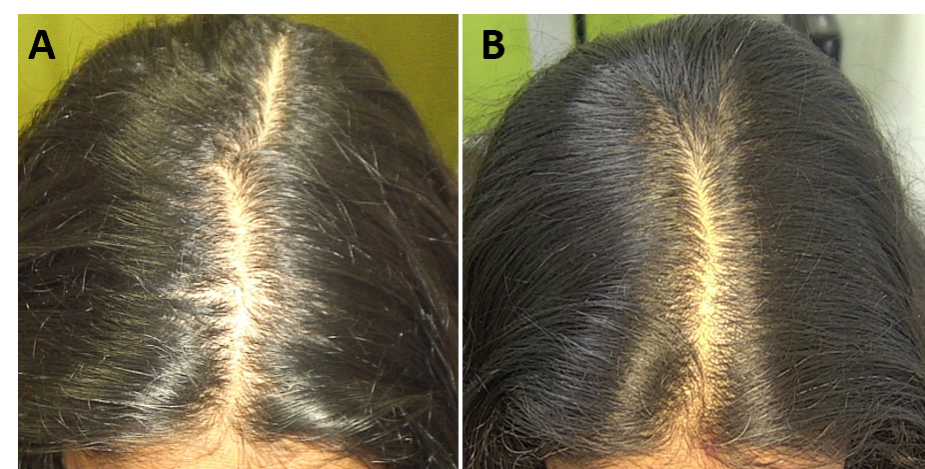
Scalp area of Indian female patient before (A) and after 14 sessions with 675-nm laser (B). A clinical improvement and restoration of the scalp’s central hairline hairs from baseline (T0) to 4-month follow-up (T1) were observed.

**Figure 4:**
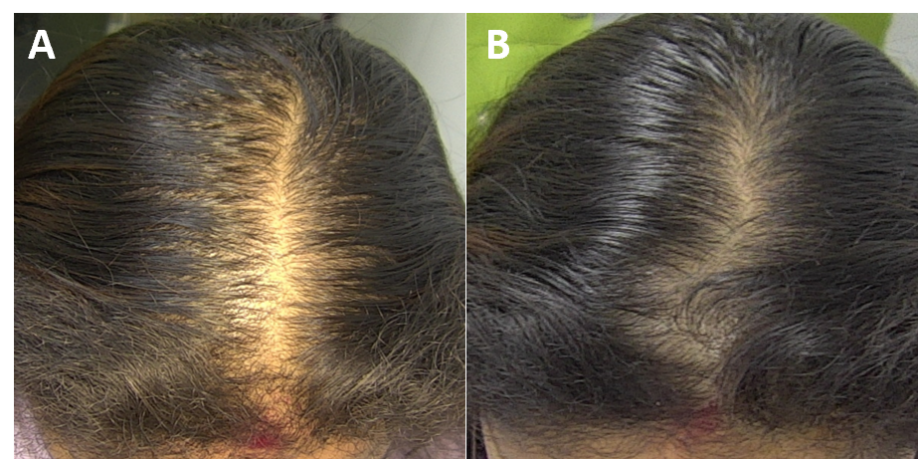
Scalp area of Indian female patient before (A) and after 14 sessions with 675-nm laser (B). A clinical improvement and restoration of the scalp’s central hairline hairs from baseline (T0) to 4-month follow-up (T1) were observed.

**Figure 5:**
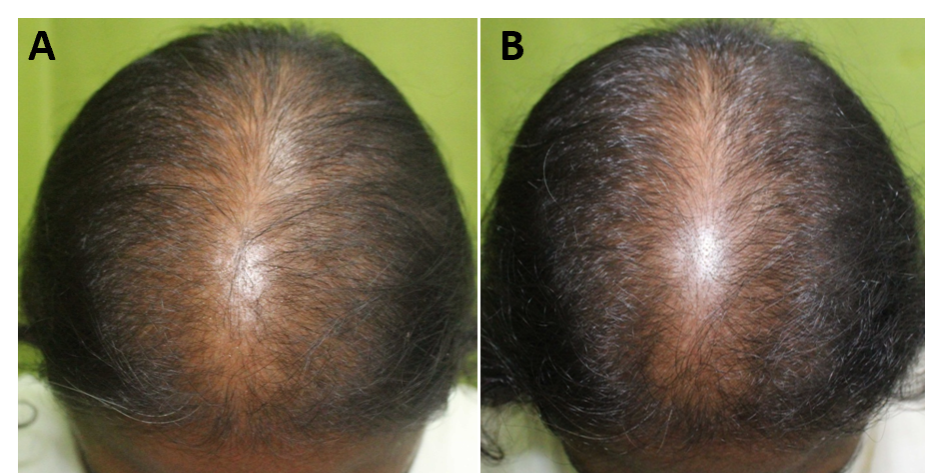
Scalp area of Indian female patient before (A) and after 14 sessions with 675-nm laser (B). A clinical improvement and restoration of the scalp’s central hairline hairs from baseline (T0) to the 4-month follow-up (T1) were observed.

**Figure 6:**
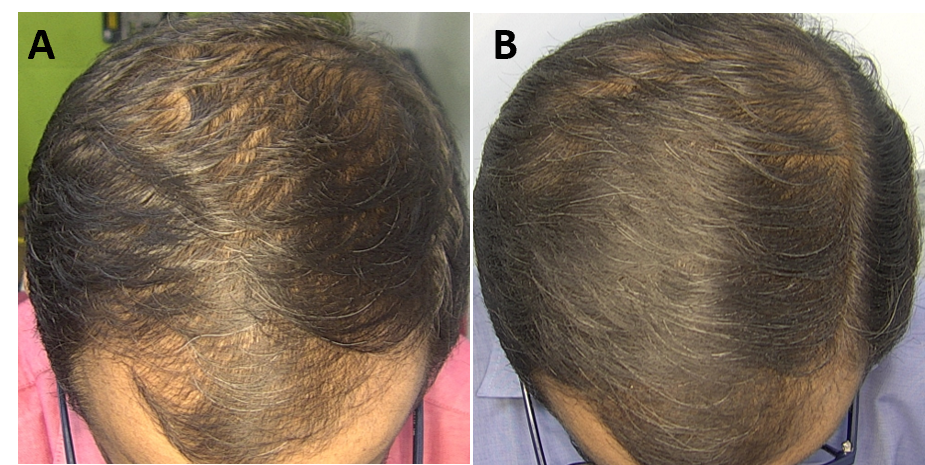
Scalp area of Indian male patient before (A) and after 14 sessions with 675-nm laser (B). A clinical improvement and restoration of the scalp’s central hairline hairs from baseline (T0) to the 4-month follow-up (T1) were observed.

**Figure 7:**
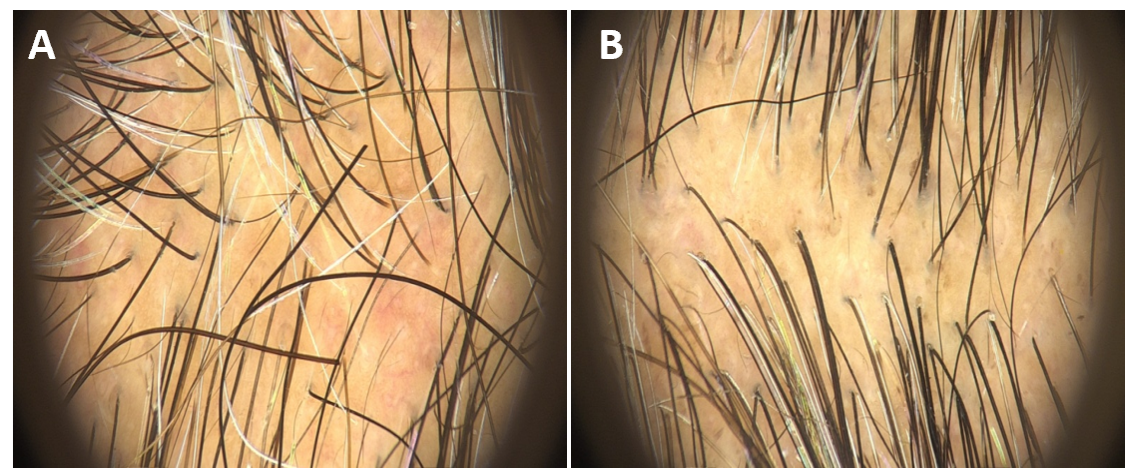
Dermatoscopy imaging of the patient’s scalp area before (A) and at the 4-month follow-up (B). A clinical improvement and restoration of the scalp’s central hairline hairs were observed.

The macro- and dermatoscopic images showed good improvement over the entire treated area, with a clear increase in the number of hairs and thickened hair (Figures 1-7). These data were confirmed by the quantitative dermatoscopic evaluation. General parameters such as hair count and hair density showed a percentage increase of around 17%.

The mean hair thickness parameters showed a significant (*P*<.001) percentage increase of 13.91%. Similar results were obtained for terminal hair and vellus hair: terminal hair count and hair density significantly (*P*=.04 and *P*=.01, respectively) increased by 17.45%, vellus hair count increased by 16.67% (*P*=.06), and vellus hair density increased by 16.61% (*P*=.06). Confirming the improvements, a significant (*P*<.001) increase in follicular units with 4 or more hairs was observed. This is a crucial aspect, since having more hair per follicular unit corresponds to greater hair density, giving the appearance of a thicker scalp, which is a sign of young and healthy hair.

## Discussion

When standard and established treatments are less effective or less productive, laser therapy offers an extra therapeutic option and is a useful adjunct to traditional therapies. For patients who are unable or unwilling to take drugs or inject platelet-rich plasma, laser therapy may be the ideal option for growing hair and preventing future progression. There are various medications available for the management of AGA. Other minimally invasive methods like the use of pulsed electromagnetic field therapy also have shown a positive biological effect on hair regrowth and were used in combination with laser therapy for the clinical treatment of AGA [34]. Among laser treatment modalities, red light, which has a skin penetration depth of 1-6 mm, improves blood circulation, promotes cell metabolism and nutrition supply to capillaries, and strengthens hair strands, anchoring follicles and pain relief (seen as an additional advantage in cases of trichodynia). The other techniques, unlike laser therapy, achieved good results but required a greater number of sessions at the same time, and the injected substances and needle pricks are not always tolerated by the patient. Red and near-infrared lasers can prolong the anagen growth phase of the HFs, promoting an increase in hair count in patients without significant side effects [35]. Indeed, laser phototherapy is assumed to facilitate anagen reentry in telogen HFs in the anagen phase, boost active anagen HF proliferation, and prevent premature catagen growth [25].

Among the laser treatment modalities, red light, with its 1- to 6-mm skin penetration depth, is the most effective in promoting cell metabolism, blood circulation, capillary nourishment delivery, cuticle anchorage, and pain relief.

The study’s findings showed that 675-nm laser technology represents a secure and efficient therapeutic approach for AGA, particularly when telogen effluvium is present. Potential mechanisms of action for this laser technology include promoting and lengthening the anagen phase of follicle hair, enhancing blood microcirculation, and stimulating fibroblasts to produce collagen and elastin.

The mechanisms through which red light acts include photobiochemical reactions with an upregulation of intracellular oxidative stress and an increment of adenosine triphosphate production (through absorption of mitochondrial protoporphyrin IX). This cellular pathway leads to an increase in reactive oxygen species and increases in transcription factors like hypoxia-inducible factor-1 and nuclear factor κB [36]. Further consequences, such as increased cell motility and proliferation; changes in the levels of cytokines, growth factors, and inflammatory mediators; and increased oxygenation of the tissue are triggered downstream by these transcription factors that regulate protein synthesis.

This mechanism has significant roles in HF growth-stimulating collagen synthesis through fibroblast growth factor activation, increasing type 1 procollagen, increasing the metalloproteinasis–9 (MMP-9) matrix, decreasing MMP-1, stimulating angiogenesis, and increasing blood flow [11]. Furthermore, published data on cultured human HFs have demonstrated that red light enhances Ki-67–positive cells, which represents a typical marker of HF cell proliferation [32].

The data reported in our study showed that there has been a 17% increase in hair length measurable parameters such as hair count, hair density, and hair thickness. Similar results were obtained for terminal and vellus hair.

Additionally, follicular units containing 4 or more hairs increased, suggesting hair revival leading to improvement in hair density and the appearance of a fuller scalp.

The study device interacts with water and the vascular component minimally while having a strong affinity for collagen and melanin. In contrast to laser systems that use wavelengths <650 nm, which are highly absorbed by hemoglobin, and wavelengths >950 nm, which are primarily absorbed by water, the wavelength of 675 nm operates directly on the collagen component based on its spectrum absorption coefficient. In this manner, the heat reaches the collagen fibers directly, bypassing other chromophores. Consequently, a thermal column was formed that diffuses heat to the surrounding areas causing immediate shrinkage and denaturation of the collagen with subsequent neocollagenogenesis [37].

Within 3 months of the treatment sessions, the qualitative and quantitative results demonstrated a significant increase in the number of vellus hairs compared with terminal hairs, indicating the revival of dormant follicles resulting in hair restoration.

The main study limitation was the small population sample and short follow-up. Additional investigations on a larger population sample will be required to standardize the criteria employed. A longer follow-up period will be expected to see whether the laser’s effects on hair growth are lasting. As a future goal, we plan to execute immunohistochemical or histological analyses.

Comparable to previous scientific research, this study looked at different parameters of accuracy like, a better hair count/density, length, thickness and follicular ratio, a photographic evaluation, and dermatoscopic analysis. Additionally, dermatoscopy helps physicians examine the skin post therapy and observe an optimal end point.

The 675-nm wavelength treatment is easy to administer and produces a minimally invasive therapy for patients, as it does not burn preexisting hair, requires no recovery period for the patient, and avoids needles and pain.

According to the research by Sorbellini et al [33], 675-nm laser treatment has proven effective in improving AGA in young patients, managing to preserve the intact epidermis and hair shaft. Indeed, the biostimulation parameters selected in this study did not damage the HFs, performing the procedure with intact hair length.

As confirmed by the results of this study and those previously published, the 675-nm laser device promises a uniform, rapid, safe, and effective method of treatment for AGA, with minimal discomfort to the patients and the potential to be combined with other treatment options.

In conclusion, the 675-nm laser system improved AGA in Indian patients, facilitating the anagen phase and improving hair density and other positive hair parameters while minimizing the risks of side effects when compared to other conventional interventions.

## Abbreviations

AGA: androgenetic alopecia
HF: hair follicle
LED: light-emitting diode
MMP-9: metalloproteinasis–9
PDT: photodynamic treatment
